# Treatment of Satoyoshi syndrome: a systematic review

**DOI:** 10.1186/s13023-019-1120-7

**Published:** 2019-06-19

**Authors:** Julián Solís-García del Pozo, Carlos de Cabo, Javier Solera

**Affiliations:** 1 Department of Internal Medicine, Hospital General de Villarrobledo, Villarrobledo, Spain; 2 Research Department, Neuropsychopharmacology Unit, Complejo Hospitalario Universitario de Albacete, Albacete, Spain; 3 Department of Internal Medicine, Complejo Hospitalario Universitario de Albacete, Albacete, Spain; 4 Department of Medical Sciences, Falculty of Medicine, Universidad de Castilla – La Mancha, Albacete, Spain; 5 Hospital General Universitario de Albacete, Unidad de Neuropsicofarmacología, Edificio de Investigación, 3ª planta, c/ Hermanos Falcó, 37, E-02008 Albacete, Spain

**Keywords:** Alopecia, Corticosteroids, Dantrolene, Diarrhea, Immunoglobulin therapy, Muscle spasms, Rare diseases, Satoyoshi syndrome

## Abstract

**Background:**

Satoyoshi syndrome is a multisystemic rare disease of unknown etiology, although an autoimmune basis is presumed. Its main symptoms are: painful muscle spasms, diarrhea, alopecia and skeletal abnormalities. Clinical course without treatment may result in serious disability or death. A review of treatment and its response is still pending.

**Results:**

Sixty-four cases of Satoyoshi syndrome were published between 1967 and 2018. 47 cases described the treatment administered. Drugs used can be divided into two main groups of treatment: muscle relaxants/anticonvulsants, and corticosteroids/immunosuppressants. Dantrolene improved muscle symptoms in 13 out of 15 cases, but not any other symptoms of the disease. Other muscle relaxants or anticonvulsant drugs showed little or no effect. 28 out of 30 cases responded to a regimen that included costicosteroids. Other immunosuppressive drugs including cyclosporine, mycophenolate mofetil, azathioprine, methotrexate, tacrolimus and cyclophosphamide were used to decrease corticosteroid dose or improve efficacy. Immunoglobulin therapy was used in nine patients and four of them obtained a favorable response.

**Conclusion:**

Corticosteroids was the most widely treatment employed with the best results in Satoyoshi syndrome. Further studies are needed to determine optimal dose and duration of corticosteroids as well as the role of other immunosuppressants and immunoglobulin therapy. Genetic or autoimmune markers will be useful to guide future therapies.

## Introduction

Satoyoshi syndrome (SS) (ORPHA 3130), also called komuragaeri disease, is a rare disorder with fewer than 70 cases reported in the medical literature. It is a multisystem disease presenting with progressive painful muscle spasms, diarrhea, endocrinopathy, alopecia, and skeletal abnormalities [1]. An autoimmune basis is likely through association with other autoimmune conditions: the presence of autoantibodies, and successful treatment of symptoms with immunosuppressants [2, 3].

The first two SS patients were described by Satoyoshi and Yamada in 1967 [4]. These authors employed multiple drugs including acetazolamide, magnesium sulfate, dexamethasone, prednisolone, diazepam, phenobarbital, diphenylhydantoin, quinine sulfate, chlorpromazine and others [4]. Despite these treatments, they failed to control muscle spasms in their patients. Eleven years later, in 1978, Satoyoshi reported 15 patients with this syndrome (including again the two first ones from 1967 [4]), most of them young women [1]. Of these 15 patients, five died, and the evolution was towards a disabling condition in the remaining patients due to failure of treatment. Since then, the existing reviews have focused on some of the manifestations of the disease [5–7], but a review of treatment and prognosis of this syndrome has not yet been carried out.

The first treatments for SS were aimed primarily to alleviate the painful and incapacitating intermittent muscle spasms. Muscle relaxants and antiepileptic drugs were used by different authors with limited results [3, 8, 9]. In the last 30 years, the drugs used for SS can be divided into two main groups of treatment: i) muscle relaxants and anticonvulsants, and ii) corticosteroids and immunosuppressants. Other treatments such as nutritional support, hormonal treatments or orthopedic surgery and rehabilitation were necessary in some cases. In the present article, we performed a systematic review of the treatment of SS.

## Material and methods

### Search strategy and inclusion of cases

All published cases of Satoyoshi syndrome were reviewed. For this purpose, a MEDLINE, Web of Knowledge (WOS), and Scopus search was performed using the keywords “Satoyoshi syndrome” or “Komuragaeri disease” with no limit for the year of publication or language. All records found up to December 2018 were included. The lists of references from the articles found by electronic search were also reviewed to identify additional records. We also reviewed the references from works cited on OMIM [10], ORPHANET [11] and Rare Diseases NIH [12] websites. All articles that reported SS cases were included.

Both the literature search and the inclusion of case reports were carried out by two of the authors. In case of disagreement between them, the final decision was reached after discussion among all the authors.

**Fig. 1:**
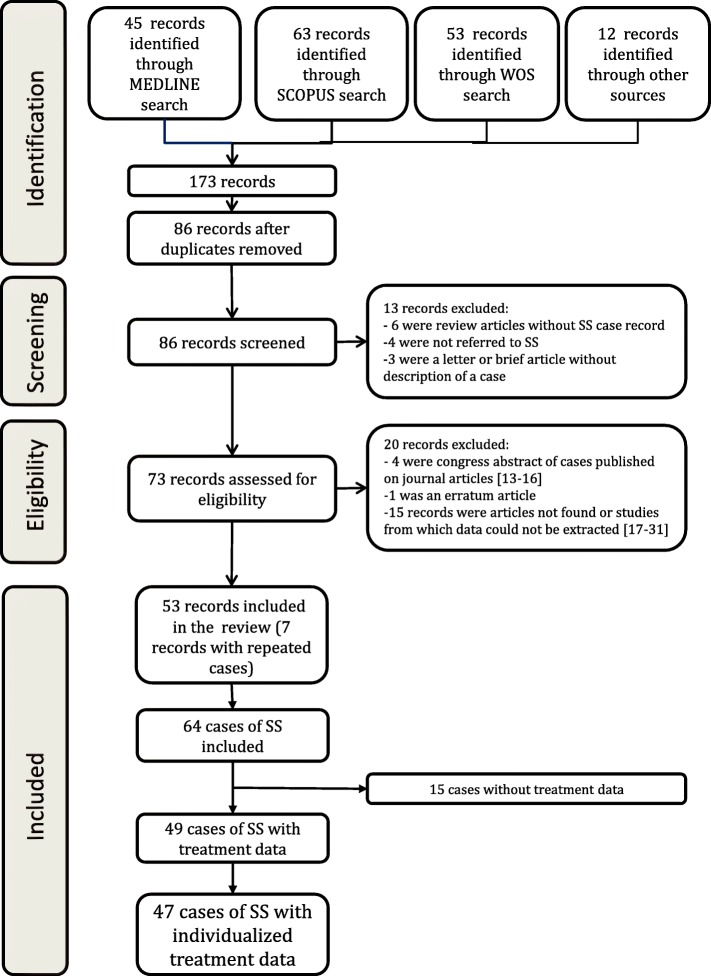
Flow chart illustrating case selection strategy [13–31]

The searches in MEDLINE, Scopus and WOS searches yielded 45, 63 and 53 articles, respectively. Twelve additional works were retrieved from reviewing the bibliographies from the articles previously found. A total of 64 cases of Satoyoshi syndrome were identified from 53 published articles (Fig. 1).

### Data extraction

The following data were extracted from each of the selected cases:

- Clinical and epidemiological characteristics: age, age at onset of symptoms and delay of diagnosis, sex, country of origin, symptoms and sings, and presence of other associated diseases.
- Treatments received including muscle relaxants, antiepileptic drugs, corticosteroids, other immunosuppressants such as azathioprine, methotrexate, mycophenolate, tacrolimus, immunoglobulin therapy or a combination of these drugs. Duration of treatment and response were also recorded.
- Outcomes: time of follow-up, mortality and sequelae.

The improvement of muscle spasms was recorded following the authors’ descriptions. This improvement was usually reported as the ability to perform the activities of daily living without significant interference from muscular symptoms. In the same way, the improvement or remission of alopecia and digestive symptoms was recorded according to the clinical case report. Usually, improvement of alopecia was considered as the regrowth of hair in the areas where it had fallen. Remission of digestive symptoms was usually described as the disappearance of diarrhea or signs of malabsorption along with weight gain. Non-response to treatment was defined as either no significant change in any of the symptoms of the disease according to the authors, or the death of the patient due to the disease. Death was considered related to SS if it was not possible to attribute it to a different cause. The time until the improvement occurred and the duration of the response were recorded if available.

### Data analysis

Data from each one of the cases were stored in an Excel database. A descriptive analysis was made after verification of the database. Qualitative data were described using frequency and percentage. Quantitative data were described as the mean ± standard deviation. Median and range were used in the case of non-normal variables.

## Results

Forty-seven out of the 64 total cases (73%) were women and 28 cases (43%) were Japanese patients, although cases of SS have been reported in other parts of the world. The age at diagnosis ranged from 5 to 65 years with a median of 16 years and with a mean of 20.3 ± 12.4 years. The average diagnostic delay was 7.5 years. Age at onset of symptoms ranged from 1 to 46 years with a median of 11 years and a mean of 13.02 ± 9.1 years. Only 13% cases of Satoyoshi syndrome were adult-onset.

All published cases had intermittent painful muscle spasms, and all had some degree of alopecia. Alopecia became universalis in 63% of cases. 37 cases (58%) had digestive alterations, mainly diarrhea. Skeletal alterations were described in 22 cases (34%) of which in 4 cases had dental occlusion problems. In 23 patients (38.3%) the presence of autoantibodies in different combinations was detected. Symptoms were progressive until onset of treatment and 7 patients died (11%). Out of the 64 patients detected, 47 [1–3, 5, 6, 8, 9, 32–68] had data on the individualized treatment administered, and in two other cases the treatment is not reported individually [4]. Seven articles described complementary data of these 49 patients [1, 69–74]. The treatment was not reported in the remaining 15 patients [1, 40, 75, 76]. There were no differences in their initial clinical characteristics between the the group of 47 SS patients whose treatment was reported and the rest of patients with SS (15 patients) whose treatment was not described in the publications (Table 1).

**Table 1:** Initial clinical characteristics of all SS patients included in this review, the 47 patients whose treatment was described and the 30 patients treated with corticosteroids

	All patients *N* = 64	Patients where treatment was specified *N* = 47	Patients treated with corticosteroids *N* = 30
Age of onset	13.02 ± 9.08	14.19 ± 10.35	13.80 ± 10.32
Female sex	47/64 (73%)	35/47 (74%)	23/30 (77%)
Muscle manifestations	100%	100%	100%
Alopecia	100%	100%	100%
Alopecia universalis	40/64 (63%)	25/47 (53%)	17/30 (57%)
Diarrhea	37/64 (58%)	26/47 (55%)	17/30 (57%)
Weight loss, low weight or growth retardation	33/64 (52%)	23/47 (49%)	14/30 (47%)
Skeletal alterations	22/64 (34%)	16/47 (34%)	10/30 (33%)
Amenorrhea	23/47 (49%)	15/35 (43%)	9/23 (39%)
ANA +	17/64 (27%)	17/47 (36%)	13/30 (43%)
Other autoantibodies	15/64 (23%)	15/47 (32%)	11/30 (37%)
Deaths	7 (11%)	2 (4%)	0 (0%)

In addition to pharmacological treatment, patients with SS have received other therapies including orthopedic surgery, rehabilitation, or nutritional treatments. However, this review will focus mainly on the pharmacological treatment.

### Anticonvulsant drugs

Phenytoin and carbamazepine were the main anticonvulsant drugs used in patients with SS (Table 2). Seven patients received treatment with phenytoin [5, 32–37]. In 4 cases phenytoin was used as the first option in combination with corticosteroids [5, 33–35]. Baclofen was also used in one of these four cases [33]. When reported, the dose administered ranged from 100 mg [34] to 200 mg daily [5, 35]. Overall, 3 out of the seven patients (42%) who received a phenytoin-containing regimen improved with this therapy [5, 34, 35], although all three cases also received treatment with corticosteroids.

**Table 2:** Non-immunosuppresive drugs used in the therapy of SS patients

Drug	Number of times used	Monotherapy as initial treatment	As initial treatment in combination with other drugs	As second or further-line treatment option	Improvement	No improvement	Change of treatment	Comments
Dantrolene	15	9	4	2	13	2	3	Dantrolene improved muscular symptoms but no other manifestations. It was used as a first option in combination with corticosteroids in two cases [6, 46] and in another case with immunoglobulin therapy [47], although, subsequently, immunoglobulin therapy was replaced by corticosteroids. Although improvement was recorded in 13 patients, such improvement only lasted for a short time in one of them, which led to the change in treatment [32]. In another case, the treatment was changed to immunoglobulin therapy because dantrolene is not a radical treatment [3]. For one of the cases that did not respond adequately, a change of treatment was not recorded in the article [45].
Carbamacepine/Oxcarbacepine	9	1	7	1	4	5	5	In two cases carbamazepine was combined with corticosteroids [2, 41]. In five cases, the treatment was changed due to lack of effectiveness [9, 32, 40, 42, 70]. In one of them, it was necessary to use botulinum toxin to control masticatory spasms [32]. In another of these cases, authors stated that carbamazepine, phenobarbital, quinine sulfate, and chlorpromazine were tested during hospitalization and they were not effective for spasms [40]. In one case, carbamazepine was used with gabapentin without result [70].
Phenitoin	7	0	4	3	3	4	1	In the 3 SS patients in whom there was an improvement, phenitoin was the first option in combination with corticosteroids [5, 34, 35]. In another patient, it was combined with blaclofen and prednisone without response [33]. In one case, phenytoin was used as a second option and no satisfactory response was obtained. But after several treatment options, phenytoin was maintained combined with corticoids and mycophenolate [37]. In another case, phenytoin was used as second option treatment with carbamazepine, but it was not effective [32]. Averianov reported its use, but without effectiveness [36].
Baclofen	3	1	2	0	0	3	3	It has been used as a first option treatment only in one SS patient [50]. In another patient, multiple muscle relaxants and anticonvulsants were used without satisfactory results [42]. Baclofen was used in combination with phenytoin and prednisone in another patient [33]. In none of the cases the treatment had a good clinical response.
Clotiapine and biperidene	1	1	0	0	0	1	1	It was not effective [51].
Clonazepam	3	0	2	1	0	3	3	Clonazepam was used in combination with carbamazepine without improvement [9] and the SS patient required changing treatment to dantrolene. Clonazepam was used in a patient adding it to dantrolene, but it was necessary to change treatment to phenytoin and carbamazepine [32]. In one patient clonazepam was used together with several other drugs such as dantrolene, carbamazepine or diazepam [42].
Tetrazepam	1	0	1	0	1	0	0	Tetrazepam in combination with carbamazepine improved spasms in a patient with SS [39].
Otilonium bromide	1	0	1	0	1	0	0	It was used in combination with carbamazepine with disappearance of spasms and diarrhea [38]
Phenobarbital	1	0	0	1	0	1	1	Phenobarbital was used in a SS patient who was also receiving carbamazepine, quinine sulfate and chlorpromazine treatment. But it was not effective either [40].
Quinine sulfate	1	0	0	1	0	1	1	Quinine sulfate was used in a patient who was also receiving carbamazepine, phenobarbital and chlorpromazine treatment. But it was not effective either [40].
Chlorpromazine	1	0	0	1	0	1	0	Chlorpromazine was used in a patient who was also receiving carbamazepine, quinine sulfate and phenobarbital treatment. But it was not effective either [40].
Acetazolamide	1	0	0	1	1	0	0	Acetazolamide improved muscle symptoms in a patient [36]. It was used in this patient after thioridazine, haloperidol and phenytoin were unsuccessful.
Neostigmine	1	0	1	0	1	0	0	Neostigmine was used in combination with traditional Chinese medicine in a patient with myasthenia. The authors stated that she remained stable after 8 months of follow-up [40].
Botulinum toxin	3	0	1	2	3	0	1	Botulinum toxin was used together with corticosteroids in a patient as a primary therapy. Treatment was changed by adding azathioprine [59]. In one case, botulinum toxin was added to the treatment to control masticatory spasms [47]. In another case, it was used after several previous treatments with dantrolene, diazepam, clonazepam, phenytoin and carbamazepine [32].
Amitriptilin	1	0	1	0	1	0	0	Amitriptilin was used in combination with corticosteroids as a maintenance therapy [61].
Thioridazine	1	1	0	0	0	1	1	[36]
Haloperidol	1	0	0	1	0	1	1	[36]
Diazepam	2	1	0	1	1	1	1	It was combined with several other treatments such as immunoglobulin therapy, cyclophosphamide and azatioprine [51]. Although this patient improved, it is difficult to attribute her improvement to diazepam. In another patient diazepam was used together with multiple muscle relaxants and anticonvulsants without satisfactory results [42].
Midazolam	1	1	0	0	0	1	0	The patient developed a neuroleptic malignant syndrome after the onset of iv midazolam and died [52].

Carbamazepine (or oxcarbazepine) were used in 9 patients with SS [2, 9, 32, 38–42, 70]. In two of them, it was used in combination with corticosteroids [2, 41]. Other drugs used in conjunction with carbamazepine as the first treatment option were otilonium bromide [38], tetrazepam [39], and clonazepam [9]. The dose was reported in four cases [2, 9, 39, 42], and ranged from 200 mg [2] to 600 mg daily [9]. Overall, 4 out of the nine patients treated with carbamazepine improved (44%) [2, 38, 39, 41], although in two of them carbamazepine was used in conjunction with corticosteroids [2, 41]. Phenobarbital was used in a patient after the failure of treatment with carbamazepine, but this treatment was not effective either [40].

### Muscle relaxants

The cases where the use of this type of drug has been reported for SS are shown in Table 2. The most frequently used drug in this group was dantrolene (15 cases) [1, 3, 6, 8, 9, 32, 33, 42–49]. Dantrolene is a muscle relaxant that disrupts calcium release from the sarcoplasmic reticulum in the skeletal muscle [77]. It has been used as a specific drug to treat malignant hyperthermia [78]. In 7 out of 13 cases of SS dantrolene dose was reported and ranged from 25 [48] to 200 mg daily [33]. Dantrolene was able to improve muscle symptoms in 13 out of 15 (87%) SS cases [1, 3, 6, 8, 9, 32, 33, 43, 44, 46–49] but it proved to be ineffective for the improvement of the other clinical manifestations of SS. In three out of the 13 cases it was used in association with corticosteroids or immunoglobulin therapy [6, 46, 47], which makes it difficult to assess the effect of dantrolene by itself.

Baclofen is a gamma-aminobutyric acid derivative that acts as a muscle relaxant mainly by disrupting polysynaptic and monosynaptic reflexes at the spinal cord level [77]. It was used in three patients with SS [33, 42, 50] without improvement in any of them.

### Benzodiazepines

Benzodiazepines were used on seven occasions: Clonazepam in 3 patients, diazepam in 2 patients, tetrazepam in 1 patient and midazolam in 1 patient. Clonazepam was always used in combination [9, 32, 42] with either carbamazepine [9], dantrolene [32], or, in one patient, with several drugs such as dantrolene, carbamazepine or diazepam [42]. None of these patients experienced clinical improvement.

Diazepam was used in combination in two patients. One patient received treatment with diazepam and other muscle relaxants without adequate response [42]. Another adult patient received treatment with diazepam in combination with other therapies such as immunoglobulin therapy and cyclophosphamide, with improvement [51].

Tetrazepam 50 mg daily was used in a 21-year-old patient along with carbamazepine 300 mg daily, with an improvement of spasms [39]. Adachi et al. [52] treated a patient with intravenous midazolam. This patient developed a malignant neuroleptic syndrome and died. The authors warned that careful attention should be paid when midazolam is used in SS.

### Systemic corticosteroids

Systemic corticosteroids are the most widely used drugs for the treatment of SS (Table 3). Out of the 47 analyzed cases, 30 were treated with systemic corticosteroids [2, 5, 6, 33–35, 37, 41, 42, 46, 47, 50, 51, 53–67]. In 22 cases the initial therapeutic regimen included corticosteroids, in 8 patients as monotherapy [53–58, 61, 63] and in 14 as combined treatment [2, 5, 6, 33–35, 37, 41, 46, 59, 60, 67]. In the remaining eight patients, corticosteroids were used after therapeutic failure of other treatments [42, 47, 50, 51, 62, 64–66].

**Table 3:** Corticosteroids and immunossuppresants drugs used in SS patients

Drug	Number of times used	Monotherapy as initial treatment	In combination with other drugs as initial treatment	As second or other treatment option	Improvement	Not improvement	Change of treatment	Comments
Systemic corticosteroids	30	8	14	8	28	2	4	In one case the treatment was changed to dantrolene for lack of efficacy [33]. In a case that had been previously treated with carbamazepine and otilonium bromide, systemic corticosteroids were prescribed for alopecia. However, treatment was interrupted a month later due to the appearance of adverse effects [13]. In one case, corticosteroids showed improvement of short duration and the treatment was changed [37]. In another case, a single pulse of corticosteroid diminished the frequency of muscle spasms during two weeks, but therapy was changed to cyclophosphamide and subsequently to azathioprine [51].
								Corticosteroids have been combined with other immunosuppressants in 7 cases. In 5 patients as initial treatment [5, 37, 60, 67] and in two patients after other treatments [50, 62]. Corticosteroids were combined with immunoglobulin therapy in two patients, [60, 67] with mycophenolate mofetil in one patient [37], with cyclosporin in two patients reported by Rudnicka [5], with methotrexate in one patient [62] and with tacrolimus in another patient [50]. Other immunosuppressants have been used in combination to low doses of corticosterois, such as methotrexate [63], azathioprine [59]. Corticosteroids have also been used in combination with other drugs such as baclofen [33], phenytoin [5, 33–35], dantrolene [6, 46], carbamazepine [2, 41] and botulinum toxin [59]. In one patient initially treated with corticosteroids in monotherapy, amitriptyline was added later [61].
Immunoglobulin therapy	9	1	3	5	4	5	6	In one case, immunoglobulin therapy was used alone as a first option without improvement [64]. In two cases, immunoglobulin therapy was used in combination with corticosteroids [60, 67]. In one case, it was used as a first option in combination with dantrolene, but treatment was changed to pulsed treatment of methylprednisolone together with dantrolene [47]. In another case, immunoglobulin therapy was used as a second option after short-term improvement with corticoids and mycophenolate mofetil, with no response [37]. In another patient they were also used as second option treatment together with diazepam [51]. Endo used them as a second option with effect for 2 months and then changing to corticoids [50]. Arita used it as second option treatment after dantrolene with satisfactory response [3]. The treatment with immunoglobulin therapy was changed in six cases [37, 47, 50, 51, 64, 69]
Cyclosporin	2	0	2	0	2	0	0	In one of the cases, both alopecia and spasms improved. In the other case, improvement of spasms was achieved, but it was less effective for alopecia [5]
Mycophenolate mofetil	1	0	1	0	1	0	1	The initial treatment in this patient was with corticosteroids and mycophenolate mofetil. Later phenytoin was added. Treatment was changed due to short-term improvement. The treatment was changed to immunoglobulin without response, and subsequently, to plasmapheresis (5 cycles) with improvement in pain and cramps. Later this same case was treated with a combination of phenytoin, mycophenolate and corticosteroids [37], thus it can be deduced that mycophenolate and corticosteroids adminstarion was maintained throughout the course of the treatment.
Azathioprine	2	0	0	2	2	0	0	It was used after treatment with both botulinum toxin and corticosteroids to help lower the dose of corticosteroids [59]. In another case it was used after multiple treatments [51]
Plasmapheresis	1	0	0	1	1	0	0	It was used after short-term improvement with both corticosteroids and mycophenolate mofetil as the first option, and failure of immunoglobulin therapy as a second option. After plasmapheresis (5 cycles) the patient improved in pain and cramps [37]
Methotrexate	2	0	0	2	2	0	0	In one case it was used together with prednisone [62], and in another case it was added to corticosteroids due to loss of effect [63]
Tacrolimus	1	0	0	1	1	0	0	It was used together with corticosteroids after trying other treatments [50]
Cyclophosphamide	1	0	0	1	0	1	1	It was used together with diazepam after trying other treatments, and was later changed to azathioprine [51]

In 16 cases corticosteroids were employed in combination with other drugs. In 9 cases, corticosteroids were used together with muscle relaxants or anticonvulsants: 3 patients with phenytoin [5, 34, 35], two patients with dantrolene [6, 46], two patients with carbamazepine [2, 41], one patient with botulinum toxin [59], and one patient with phenytoin and baclofen [33]. In the 7 other patients, corticosteroids were used in combination with other immunosuppressants: two patients with cyclosporin [5], one patient with mycophenolate mofetil [37], one patient with methotrexate [62] and two patients with immunoglobulin therapy [60, 67] and one patient with tacrolimus [50].

In another two cases, immunosuppressants were subsequently used to lower the dose of corticosteroids in two patients (methotrexate in one patient [63] and azathioprine in another one patient [59]).

The corticosteroid drugs used were prednisone (12 patients) [2, 5, 33, 41, 55, 56, 59–62, 64], prednisolone (12 patients) [6, 34, 35, 37, 42, 50, 54, 58, 63, 65–67],methylprednisolone (6 patients) [37, 42, 46, 47, 50, 51] and triamcinolone (in one case) [5]. Two additional patients were treated with corticosteroids without specifying the drug used [53, 57]. In five patients, methylprednisolone was administered as intravenous boluses at a high dose [42, 46, 50, 51, 63] during a period of 3 days that could be extended for up to 4–6 weeks [63]. The doses of oral corticosteroids ranged from 2 mg / kg / day of prednisolone [65] to 0.3 mg / kg / day of prednisone [5], with subsequent dose reductions.

Taken together, 28 out of 30 patients (93%) responded to a regimen that included corticosteroids. The optimal treatment duration could not be clearly determined, since in most of the published clinical cases the follow-up time was limited. That notwithstanding, improvement was reported at two or more years of follow-up [35, 46].

### Other immunosuppressive drugs

Other immunosuppressive drugs including cyclosporine, mycophenolate mofetil, azathioprine, methotrexate, tacrolimus and cyclophosphamide, were used in 9 cases for the treatment of SS. In eight patients they were used in combination with corticosteroids. Table 3 reports the number of times these drugs have been tested in the treatment of SS.

Cyclosporine at a dose of 50 mg daily was used in two patients (two mg/kg/day in one of the patients and 3.33 mg/kg/day in the other) in combination with prednisone [5]. Both patients showed improvement of the spasms and only one of them had an improvement in alopecia.

Azathioprine was also used in two cases. In one of them, azathioprine was used in monotherapy after having tried other treatment options that included clotiapine, biperiden, cyclophosphamide, diazepam, immunoglobulin therapy and a 3-day cycle of high doses of methylprednisolone [51]. In the other case, azathioprine was prescribed to lower corticosteroid doses due to side effects [59].

Methotrexate was another drug from this group that was used for two patients. In an adult patient it was used at a dose of 7.5 mg weekly together with 30 mg daily of prednisone, resulting in improvement of all symptoms within weeks, excepting alopecia [62]. In the other case (a 14-year-old girl), methotrexate was added to corticosteroids at a dose of 10 mg/m2 once a week to enhance effects, and reduce the dose of corticosteroids [63].

Mycophenolate mofetil was used in a 30-year-old patient [37], originally together with corticosteroids. After an initial response, the patient worsened and treatment with phenytoin was added. Because of the poor control of symptoms, treatment with immunoglobulin therapy was tried, but also without success. Later plasmapheresis was prescribed (5 cycles), improving cramps and pain. As a maintenance treatment, the patient continued with corticoids, mycophenolate, and phenytoin.

### Intravenous human immunoglobulin therapy

Immunoglobulin therapy was used in 9 cases [3, 37, 47, 50, 51, 60, 64, 67, 69] and it was the second most frequently used immunosuppressive treatment after corticosteroids. In 4 cases, treatment with immunoglobulin therapy was part of the initial treatment of patients with SS [47, 60, 64, 67]. Only in one case immunoglobulin therapy was used in monotherapy as the first therapeutic option, but no improvement of the patient was achieved [64]. In 3 other patients, immunoglobulin therapy was used as initial treatment in combination with either corticosteroids (2 patients) [60, 67] or dantrolene (1 patient) [47]. In five patients they were not used as part of the initial treatment regimens [3, 37, 50, 51, 69]. In one of these cases immunoglobulin therapy were added after treating the patient with corticoids, mycophenolate mofetil and phenytoin, without showing efficacy [37]. Another case was an adult woman in whom immunoglobulin therapy were a second treatment option in combination with diazepam [51]. In this patient, the effect of a 5-days cycle of immunoglobulin therapy was beneficial in the improvement of muscular spasms for 6–8 weeks. After 2 cycles, immunoglobulin therapy was stopped and changed to cyclophosphamide, as her medical insurance company was unwilling to pay for additional immunoglobulin therapy cycles. In three other patients, immunoglobulin therapy were used as monotherapy after having tested dantrolene (1 patient) [3] and baclofen (1 patient) [50] or carbamacepine and gabapentin (1 patient) [69, 70]. In the first two cases, both patients improved but in one of the cases the improvement was brief, and treatment changed to corticoids [50]. The third patient did not improve [69]. In summary, only 4 out of the 9 patients treated with immunoglobulin therapy, obtained some degree of favorable response (44%).

### Other treatments

Botulinum toxin was employed in three patients for controlling masticatory muscle spasms [32, 47, 59]. In one of them, botulinum toxin was used injected into both masseter muscles to control the trismus as a first treatment option together with systemic corticosteroids [59]. Merello et al. reported the use of botulinum toxin because of poor control of spasms with other treatments such as dantrolene, diazepam, clonazepam, phenytoin and carbamazepine [32].

Muscle massage together with analgesics such as paracetamol, did not achieve any improvement [65]. Techniques of traditional Chinese medicine were used together with neostigmine in a patient with SS and myasthenia. The authors reported that after 8 months the patient was stable [40].

Topical corticosteroid treatment was tested in three patients with alopecia, without beneficial results [5, 13, 64]. There is only one case reporting a response to diphencyprone, a drug used for alopecia areata [13]. Kamat et al. reported a patient who started treatment with minoxidil followed by topical steroids after he began losing hair on his scalp. Despite this treatment, he continued experiencing hair loss on his scalp [64]. Another patient reported by Ashalata et al., tried the treatment with minoxidil before the diagnosis was made, but without favorable result [35]. In one case, UVB rays were used to try to improve alopecia, but also without result [13].

For the control of diarrhea, a diet with restriction of simple carbohydrates was tried without results [2]. In another case with significant digestive manifestations, parenteral hyperalimentation was administered with weight improvement but without resolution of diarrhea, amenorrhea or alopecia [68]. Subsequently, this patient suffered episodes of recurrent pancreatitis attributed to stenosis of the duodenal papilla due to fibrosis of the duodenal mucosa. Gastrojejunostomy, percutaneous enterostomy, and percutaneous cholangiostomy were performed. The patient died a few months later due to sepsis [68]. This patient did not received therapy with corticosteroids or immunosuppressants.

In one case, the authors comment that treatment with estradiol and norgestrel was started to achieve regular menstrual cycles as well as breast development [63]. Growth hormone was also used to achieve greater growth [38]. In some patients, orthopedic surgery was necessary due to skeletal alterations [35, 65, 73].

### Prognosis

Since the introduction of corticosteroids in the treatment, the prognosis of patients with Satoyoshi syndrome has improved. We found seven patients who died due to SS in the literature search [1, 52, 68]. Five out of these seven cases were described by Satoyoshi in 1978 [1]. The two other cases were those described by Nagahama et al. [68] and by Adachi et al. [52]. The first one was a patient with digestive manifestations and lesions compatible with cystic gastroenteritis. He died due to sepsis after suffering several episodes of recurrent pancreatitis and undergoing biliary and gastrojejunal surgery. The second case died as a consequence of a neuroleptic malignant syndrome after the start of treatment with intravenous midazolam. Only two one of the seven cases who died could have received corticoids at some point.

Regarding the clinical manifestations of the syndrome, as already mentioned, muscular symptons improve in most cases with corticoids or dantrolene and the patient was able to carry on with a normal life with little interference from symptoms [2, 6, 35, 41, 61, 65, 73]. A smaller percentage of patients was able to recover from alopecia. Although hair regrowth was reported in some cases, complete full hair recovery was rare [2, 5, 6, 35, 41, 61, 65, 73]. Digestive symptoms also responded to treatment with steroids, with disappearance of diarrhea [2, 41, 46]. Menstruation also reappeared in many of the patients [35, 41, 66, 73].

## Discussion

Our review suggests that the best treatment for SS was corticosteroids administration. These drugs have been the primary treatment that has allowed an improvement in the prognosis of this disease. This improvement in prognosis is reflected in the fact that after the cases reported by Satoyoshi, mortality has been nil in the cases that received corticoid treatment. However, the appropriate duration of treatment, best corticosteroids dose, or the indication and time to add other immunosuppressive drugs, are still unknown. Other immunosuppressive drugs have been scarcely used, and most of the times they were administered in association with corticosteroids to reduce their dose or avoid adverse effects. Thus, it is not currently known whether their addition to corticosteroids allows increasing the efficacy of the treatment. Anticonvulsants and muscle relaxants were widely used in the first patients described [32, 33, 42, 49]. These drugs have not shown to be effective. In general, patients that improved with these drugs also received therapy with corticosteroids [2, 5, 34, 35, 41, 46, 47], therefore making difficult to assess the actual improvement of symptoms they cause. Only dantrolene showed efficacy in controlling muscle manifestations, but it failed to improve other symptoms of SS. Also, the management of SS includes not only pharmacological treatment but also other therapeutic approaches such as splints, botulinum toxin, dental procedures, surgery and orthopedic therapies and rehabilitation.

Among the limitations of this review are that it is based on case reports with a small number of patients, sometimes with an incomplete description and with a short follow-up. As with other rare diseases, there are no treatment guidelines or recommendations based on comparative studies. However, the review of the literature points towards a combination of immunosuppressant drugs based on corticosteroids. In addition, because only a few patients have been followed in the long-term, it is not possible to make recommendations about the duration of therapy or the rate of reduction of corticosteroids over time. On the other hand, the recorded response to treatment in SS patients was mainly clinical. There are no biological markers to predict or monitor the effect due to the medication.

Clinical experience supports the probable association between autoimmunity and Satoyoshi syndrome. In the next years, it is likely that further research may determine the role of specific autoantibodies in the pathogenesis and help the management of Satoyoshi syndrome. The discovery of the presence of antibodies against brain [75, 79] and gastrointestinal tissue [75] by means of western blot, opens a way to identify specific autoantibodies related to the pathogenesis of this syndrome which may become a diagnostic tool in the future.

On the other hand, the study of familial aggregation and possible genetic component of this disease is hampered by a lack of reports on the descendants of affected patients. The fact that amenorrhea or uterine hypoplasia are among the possible manifestations in women with SS make it difficult for these patients to have offspring. The association of SS with an autosomal recessive inheritance pattern [62] opens a new avenue of research in this field.

Another challenge is to achieve the collaboration among the different specialists who have treated SS patients, and particularly, the creation of an international registry of SS cases. Data from this future international registry should help to correlate the genetic and autoimmune information with the clinical characteristics and response to treatment.

## Conclusions

Satoyoshi syndrome is a rare disease with characteristic manifestations that make its clinical diagnosis easy if it is suspected. Since its description in the decade of the 60s, a multitude of drugs have been tested for its treatment. Our review suggests that the best treatment for SS was corticosteroids administration. Corticosteroids were the most widely used type of drugs (with different regimens, dosages and formulations), with the best results. However the differences in treatments, impaired follow-up data and small number of cases prevents any definitive conclusions. The use of corticosteroids and immunosuppressants has improved prognosis significantly. Other than corticosteroids and immunosuppressants, the drug that obtained the best response in the control of muscle spasms was dantrolene. This drug can be used in conjunction with corticosteroids or other immunosuppressants, although it has failed to show effect in non-muscular manifestations.

Pending issues are: the optimal treatment duration to achieve a sustained response with minimal side effects, the optimal dose of corticosteroids to be used, or whether the use of high dose intravenous boluses of corticosteroids every 4 to 6 weeks is better than daily oral doses. It is not clear either whether the combined use with methotrexate, azathioprine or cyclosporine is an alternative that will allow reducing or suspending corticoid treatment after a certain period of time.

SS is a complex and multisystemic disease. The approach to patients must be individualized according to the patient’s manifestations, requiring a multidisciplinary team for their management. As it happens in other rare diseases, only data sharing and coordinated research among different clinical and research groups can lead to results that improve the clinical management of SS patients.

## Data Availability

Not applicable.

## Abbreviations

SS: Satoyoshi Syndrome
WOS: Web of Science
